# The ideal couch tracking system—Requirements and evaluation of current systems

**DOI:** 10.1002/acm2.12731

**Published:** 2019-09-19

**Authors:** Alexander Jöhl, Stefanie Ehrbar, Matthias Guckenberger, Stephan Klöck, Andreas Mack, Mirko Meboldt, Melanie Zeilinger, Stephanie Tanadini‐Lang, Marianne Schmid Daners

**Affiliations:** ^1^ Product Development Group Zurich Department of Mechanical and Process Engineering ETH Zurich Zurich Switzerland; ^2^ Department of Radiation Oncology University Hospital Zurich Zurich Switzerland; ^3^ University of Zurich Zurich Switzerland; ^4^ Institute for radiotherapy Klinik Hirslanden Zurich Zurich Switzerland; ^5^ Institute for Dynamic Systems and Control Department of Mechanical and Process Engineering ETH Zurich Zurich Switzerland

**Keywords:** intrafractional motion, motion compensation, treatment couch tracking, robotic couch

## Abstract

**Introduction:**

Intrafractional motion can cause substantial uncertainty in precision radiotherapy. Traditionally, the target volume is defined to be sufficiently large to cover the tumor in every position. With the robotic treatment couch, a real‐time motion compensation can improve tumor coverage and organ at risk sparing. However, this approach poses additional requirements, which are systematically developed and which allow the ideal robotic couch to be specified.

**Methods and materials:**

Data of intrafractional tumor motion were collected and analyzed regarding motion range, frequency, speed, and acceleration. Using this data, ideal couch requirements were formulated. The four robotic couches Protura, Perfect Pitch, RoboCouch, and RPSbase were tested with respect to these requirements.

**Results:**

The data collected resulted in maximum speed requirements of 60 mm/s in all directions and maximum accelerations of 80 mm/s^2^ in the longitudinal, 60 mm/s^2^ in the lateral, and 30 mm/s^2^ in the vertical direction. While the two robotic couches RoboCouch and RPSbase completely met the requirements, even these two showed a substantial residual motion (40% of input amplitude), arguably due to their time delays.

**Conclusion:**

The requirements for the motion compensation by an ideal couch are formulated and found to be feasible for currently available robotic couches. However, the performance these couches can be improved further regarding the position control if the demanded speed and acceleration are taken into account as well.

**Abbreviations**
AP: anterior–posterior
LR: left–right
SI: superior–inferior

## 1 INTRODUCTION

Radiotherapy precisely focuses the ionizing radiation on the tumor volume. However, motion induced by respiration during treatment delivery causes substantial uncertainty. The peak‐to‐peak motion amplitudes of lung and liver tumors are reported to amount to 38^1^ and 34 mm,^2^ respectively. If this motion is not adequately taken into account, the effectiveness of the treatment is reduced considerably.^3^

The established approach to account for intrafractional motion thus far has been to expand the target volume, such that it covers the intrafractional motion of the tumor at the planning CT. While this approach assures the tumor’s radiation dose coverage, it does increase the irradiation dose to healthy tissue surrounding the tumor. Several approaches were proposed to overcome the uncertainty due to intrafractional motion,^4^ which aim at reducing the dose to healthy tissue while ensuring the full dose coverage of the tumor.

1. During gated treatment, the motion of the tumor is observed and the radiation beam is only switched on if the tumor is in a specific position.^4^
2. With the Cyberknife^5^ or the Vero^6^ system, the tumor motion is continuously compensated by moving the beam.
3. With multileaf collimator (MLC) tracking,^7^ the beam position and shape are modified with the MLC in accordance with the tumor movement. This approach not only could compensate for changes in tumor position but also for changes in tumor shape.
4. During couch tracking, the patient is moved with the robotic couch to keep the tumor in the beam. These approaches were compared and were found to perform similarly.^8^

Traditionally, the robotic couches have been designed and used for the static positioning of the patient to correct for setup errors. Since the robotic couches can move continuously, they have been investigated regarding their use for couch tracking. Several robotic couches have been evaluated for couch tracking.^9^, ^10^, ^11^, ^12^, ^13^, ^14^, ^15^ All investigations used different motion trajectories, which makes a comparison of the systems difficult. Additionally, in all these cases, the performance testing relied on motion trajectories, which were restricted to either sinusoidal motion trajectories with a limited number of amplitudes and frequencies or to a limited number of patient‐derived motion trajectories. Thus far, no systematic evaluation has been conducted with chirp signals.

The ideal robotic couch has to be able to move according to the characteristics of tumor motion such as speed or acceleration to compensate successfully for tumor motions that occur in clinical practice. The requirements for such a robotic couch thus have to be determined such that the performance of current couches with respect to these requirements can be investigated. Four robotic couches were tested: the Protura (CIVCO Medical Solutions, Kalona, IA, USA), the Perfect Pitch (Varian Medical Systems, Palo Alto, CA, USA), the RoboCouch (Accuray Inc., Sunnyvale, CA, USA), and the RPSbase (gKteso GmbH, Bobingen, Germany). A custom measurement system was designed that measured the positions as well as the orientations of the couches during the performance tests. The data obtained from these performance tests led to recommendations for robotic couches regarding their active motion compensation.

This work aimed at a systematic development of requirements for ideal treatment couches used in active motion compensation, an evaluation of current robotic couches with respect to the requirements derived, and a recommendation for modifications of current robotic couches or a new couch design for an ideal couch tracking system.

## 2 METHODS AND MATERIALS

### 2.1 Tumor motion characteristics

To estimate the tumor motion characteristics, two main types of information sources were used: First, data reported in literature and second, measured tumor motion trajectories.

Data^2^, ^4^, ^16^ on tumor motion were analyzed, followed by analysis of all references as well as publications citing these studies. This analysis yielded a comprehensive compilation of studies reporting data on tumor motion. The publications were examined for values reported per tumor motion trace. These values were included with respect to the motion direction. If no directions were stated, the values reported were included in every direction. The measured tumor motion trajectories were analyzed regarding the displacement by independently computing the 1%–99% range of measured positions in the superior–inferior (SI), left–right (LR), and anterior–posterior (AP) directions. Analogously, the data were differentiated to obtain the speed and acceleration of these signals, and the 99^th^ percentiles of the absolute speed and acceleration signals were taken. Using these percentiles instead of maximal values is more robust against measurement uncertainties such as outliers. The data were analyzed regarding the respiration frequencies by computing the peak‐to‐peak time intervals and taking their median. The tumor motion data formed the basis for determining the requirements for a robotic couch to be used for couch tracking. An overview of the data collected is shown in Table 1.

**Table 1:** Overview of data included in the characterization of tumor motion.

	Data reported in literature		Tumor motion trajectories analyzed	
	Location	References	Data	Published in
Displacement	Liver, lung, right kidney, left kidney, diaphragm	2, 16, 17, 18, 19	Lung motion	20, 21
			Prostate motion	22, 23
Speed	Liver, lung, kidney	24, 25	Lung motion	20, 21
			Prostate motion	22, 23
Acceleration	Liver, kidney	24	Lung motion	20, 21
			Prostate motion	22, 23
Frequency	Liver, lung, kidney, abdomen	16, 17, 19, 26, 27	Lung motion	20, 21
			Respiratory external motion	28

### 2.2 Robotic couch characteristics

The four treatment couches Protura, Perfect Pitch, RoboCouch, and the RPSbase were investigated. The Protura is a robotic couch that is fixed on a pedestal, as shown in the supplemental materials. Its manufacturer CIVCO specifies the translational positioning to reach submillimeter accuracy.^29^ The mechanics are based on parallel kinematics, which guarantee a high stiffness and accuracy.^15^ The Protura is available on the market and is in clinical use. The Perfect Pitch by Varian is specified to reach a positioning accuracy of 0.5 mm.^30^ Its mechanics are based on serial kinematics with scissor kinematics for the vertical direction and with belts in the lateral and longitudinal directions. The Perfect Pitch is also available on the market and is in clinical use as well. The RoboCouch by Accuray is offered in combination with the manufacturer’s Cyberknife system. Its repeatability is stated to be 0.1 mm,^31^ and its mechanics are based on serial kinematics with revolute joints (like robots for the automated production of automobiles). For the RPSbase, gKteso specifies an accuracy of 0.1 mm for corrective motion and 0.5 mm for absolute positioning.^32^ Its mechanics are based on scissor kinematics, and it is available on the market as well.

**Figure 1:**
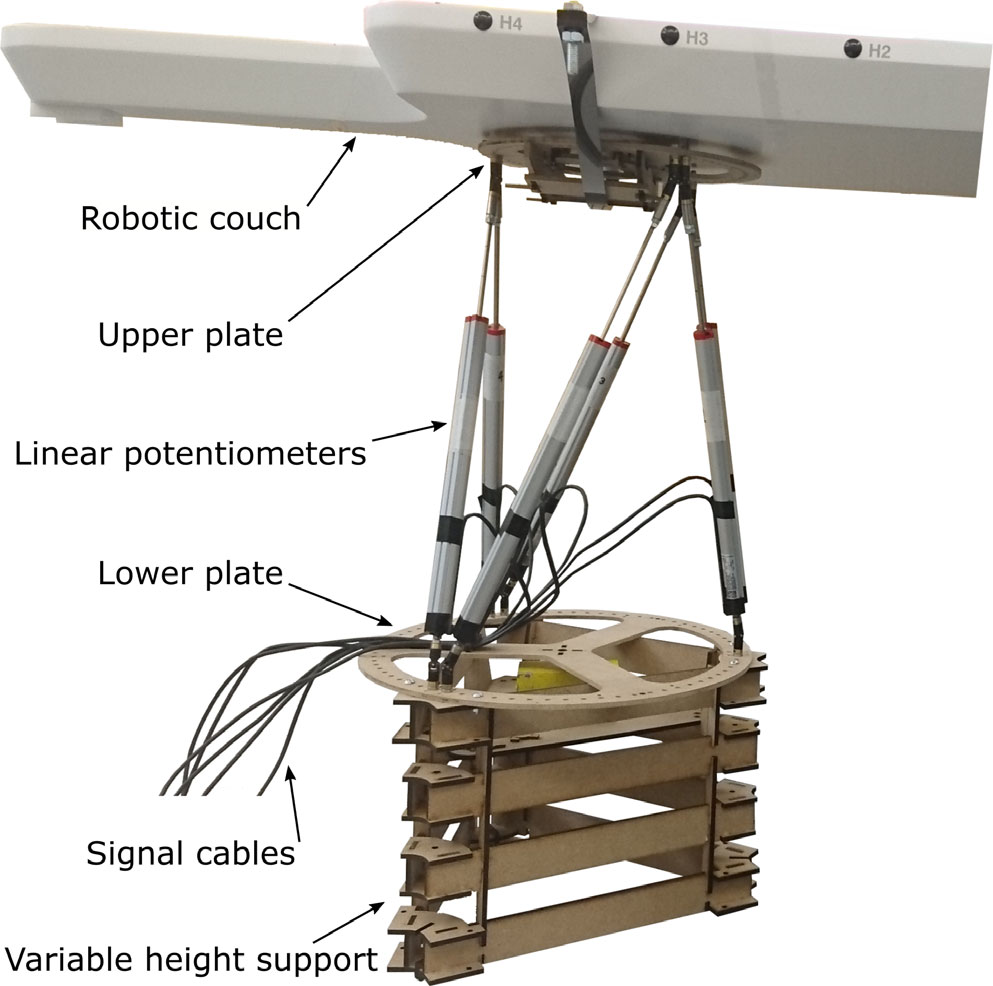
Measurement device consisted of a lower plate and an upper plate connected by six linear potentiometers in parallel. The upper plate was fixed to the robotic couch. The lower plate was fixed to the support, which was placed on the ground. The height of the support could be varied to accommodate the measurement system for different robotic couches.

For determining the specific characteristics of the four robotic couches, a number of performance tests were designed. For all these tests, weights were placed on the couches and positioned such that they mimicked a patient placed in a supine position and weighing 98 kg. The positions of the weights were determined by considering the relative weights of the body parts^33^ and their locations, as shown in the supplemental materials. The measurement device consisted of six linear potentiometers (Opkon, Istanbul, Turkey), which were arranged in parallel between two plates, see Fig. 1. During the performance tests, the measurement device was positioned beneath the couch plate, as shown in the supplemental materials. The lower plate was fixed to the ground, while the upper plate was attached to the couch. The choice of the linear potentiometers was motivated by their mechanical design that integrates the sensor and the linear bearing that restricts the sensor’s motion to one dimension. This integrated design enabled the design of the entire measurement device, which aimed at the ability to simultaneously measure all three translational degrees of freedom as well as all three rotational degrees of freedom.

The design of the measurement device ensured that the position and orientation of the upper plate relative to the lower plate corresponded directly to the lengths of the potentiometers. The signals of the potentiometers thus could be used to compute the position and orientation of the couch. The analog output signals of the potentiometers were sampled at 500 Hz. The measurement device was tested with the Hexapod H840.5PD (Physik Instrumente GmbH & Co. KG, Karlsruhe/Palmbach, Germany), which moved to points in a predefined three‐dimensional grid and paused at each point for at least one second. The measurement device’s standard deviations of the translational errors were 0.12 mm in the longitudinal, 0.13 mm in the lateral, and 0.06 mm in the vertical direction. The standard deviations of the rotational errors were 0.02° around the longitudinal direction, 0.02° around the lateral direction, and 0.03° around the vertical direction. Further results are detailed in the supplemental materials.

#### 2.2.1 Types of performance tests

Six different tests were performed on each of the four robotic couch systems.

1. Motion range tests, the maximum values found in the tumor displacement data collected determined the motion range requirements of the couches. As shown in the Results section below, these limits amounted to 59.9 mm longitudinal, 36 mm lateral, and 30.2 mm vertical. The couches had to move at least in these required ranges.
2. Static accuracy tests, the robotic couches moved to 268 distinct coordinates inside the range requirements. At each point, the couches waited for at least one second such that the errors in the longitudinal, lateral, and vertical directions could be computed. Typically, the spatial resolution of a computed tomography image is around 0.24 mm, while the slice thickness varies from 0.5 to 10 mm. Therefore, the ideal static accuracy of a robotic couch should be below 0.24 mm.
3. Maximum speed tests, the robotic couches repeatedly moved back and forth between two points located at least 20 mm apart in the longitudinal, lateral, or vertical direction. At both points, the couches waited for at least one second before returning. The measured position signal was differentiated and the peaks of the resulting speed signals indicated the maximum speeds.
4. Maximum acceleration tests, the robotic couches moved back and forth between two points in the longitudinal, lateral, or vertical direction, but the couches immediately reversed their direction when they reached their end point. The measured position signal was differentiated twice and the peaks of the resulting acceleration signal indicated the maximum accelerations.
5. Time delay tests, the robotic couches followed slow sinusoids with a sinusoidal constant frequency of 0.05 Hz. The resulting position measurement signal was shifted in time, which then was quantified using a cross correlation of the measurement signal and the input signal. The test of the Accuray RoboCouch had to be treated differently as the input signal and the position measurement signal could not be synchronized. Instead, the time from releasing the motion‐enable button until the peak deceleration of the couch could be measured as a representative of the time delay.
6. Chirp signal tests, the robotic couches followed chirp signals in the longitudinal, lateral, or vertical direction. The chirp signal frequency continuously increased from 0.1 to 0.5 Hz. Additionally, the chirp signals were applied repeatedly with varying peak‐to‐peak amplitudes of 4 to 60 mm in the longitudinal, 4 to 40 mm in the lateral, and 4 to 40 mm in the vertical direction, respectively. The difference between the measured position and the chirp signal was computed and was denoted as tracking error. The ratio of the Fourier transformations of the tracking error and the chirp signal then was computed. This ratio’s magnitude yields the normalized residual motion as a function of motion frequency.

## 3 RESULTS

### 3.1 Tumor motion characteristics and couch performance requirements derived

**Figure 2:**
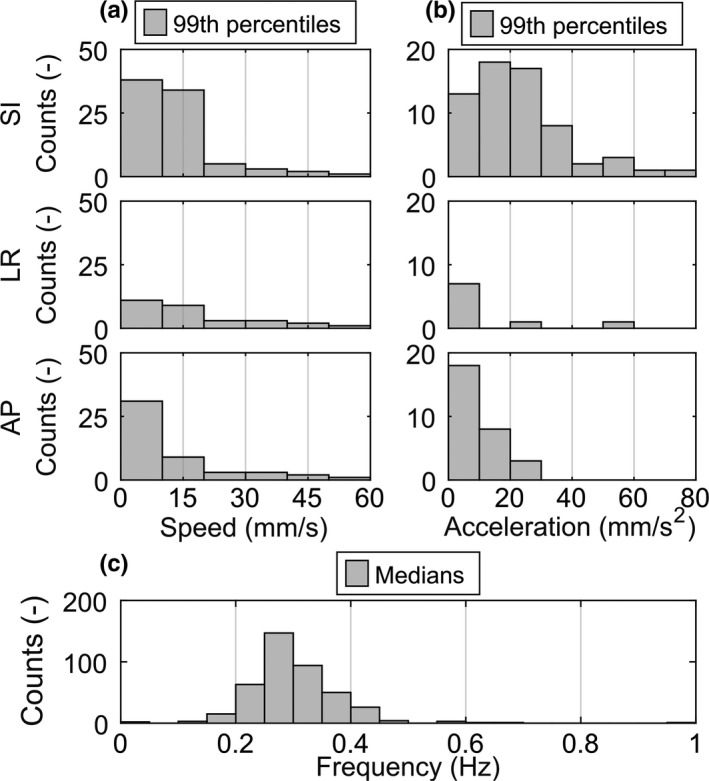
(a) The left histograms show the speed and (b) the right histograms the acceleration in the superior–inferior (SI), the left–right (LR), and the anterior–posterior (AP) directions. (c) The distribution of the tumor motion frequency. The data were obtained from the sources listed in Table 1. For each measured respiratory motion trace, the 99^th^ percentiles of the speed and the acceleration as well as the median of the frequency were computed.

The assessment of the data collected from literature combined with the results of the data analysis showed a median peak‐to‐peak motion of 11.8 mm with a maximum of 59.9 mm in the SI, 5 mm with a maximum of 36 mm in the LR, and 4.5 mm with a maximum of 30.2 mm in the AP directions, respectively. The distribution of the speed is shown in Fig. 2(a), while the Fig. 2(b) shows the acceleration and panel c) depicts the frequency.

The motion data of all the tumors formed the basis for determining the requirements for a robotic couch to be used for couch tracking. Assuming the patient to be placed in a supine position, the direction of the tumor SI motion corresponds to the longitudinal direction, while LR corresponds to the lateral, and AP to the vertical direction of the couch motion. Based on the speed data shown in Fig. 2(a), the robotic couches are required to reach maximum speeds of 60 mm/s longitudinally, 60 mm/s laterally, and 60 mm/s vertically, respectively. Analogously, the acceleration data shown in Fig. 2(b) results in defining the maximum accelerations at 80 mm/s^2^ longitudinally, at 60 mm/s^2^ laterally, and at 30 mm/s^2^ vertically. Given the peak‐to‐peak motion data, the robotic couches are required to attain ranges for motion compensation of at least 60 mm longitudinally, 40 mm laterally, and 40 mm vertically, respectively. Finally, the motion frequency data shown in Fig. 2(c) set the requirement that the couches should be able to track periodic motions of up to 0.5 Hz.

## 4 Robotic couch characteristics

**Figure 3:**
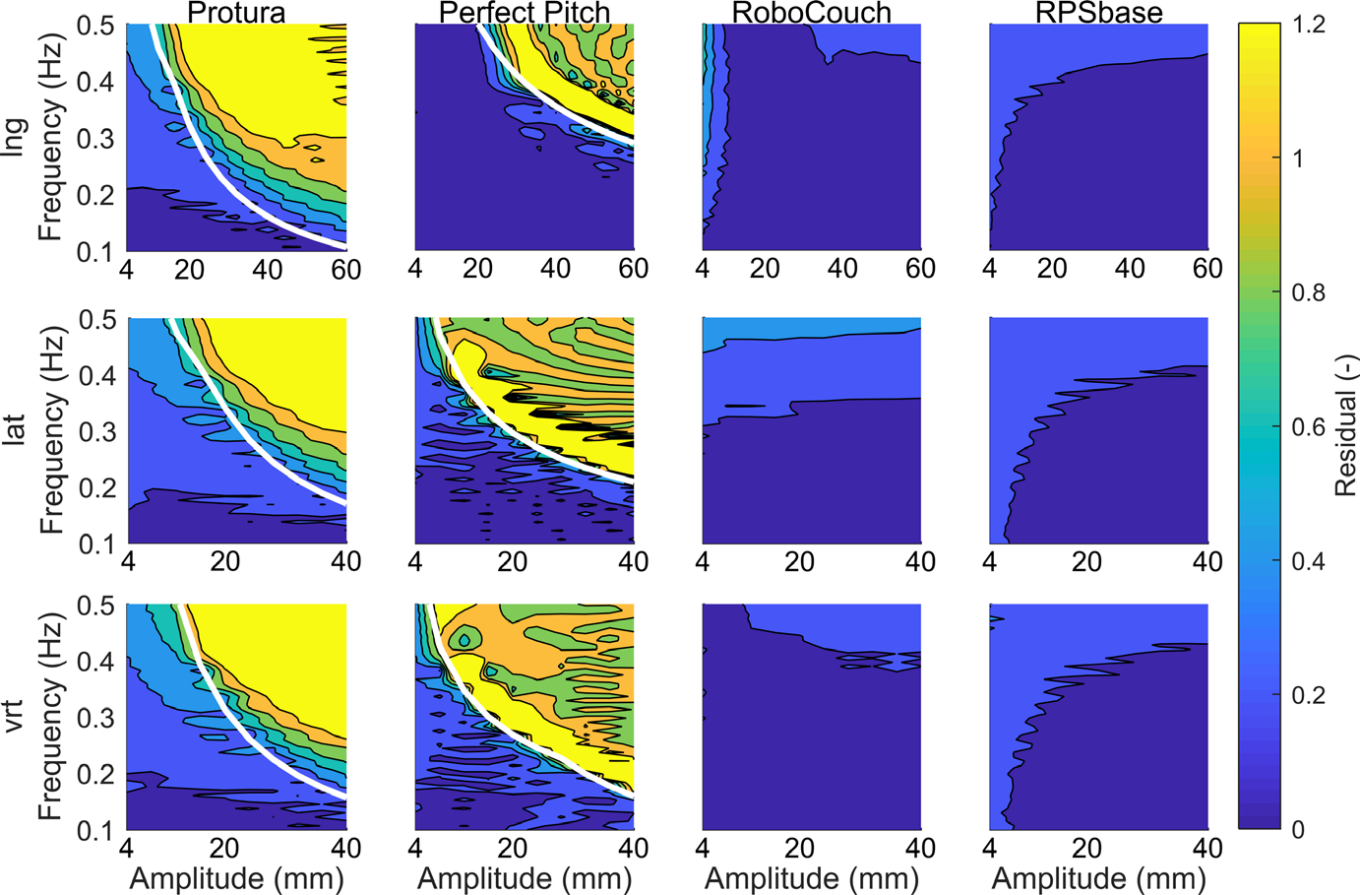
For each robotic couch, the residual motion (normalized by input amplitude) is shown for a chirp signal input (sinusoidal with continuously increasing peak‐to‐peak amplitude from 4 to 60 mm in longitudinal [lng], 4 to 40 mm in lateral [lat], and 4 to 40 mm in vertical [vrt] directions). The frequencies varied from 0.1 to 0.5 Hz. The *white lines* indicate the border above which the maximum speeds and acceleration of the couch were lower than the maximum speeds and acceleration of the input motion.

The following six tests were performed on the robotic couches:

1. Motion range tests shown in Table 2. All four couches can attain the peak‐to‐peak tumor motion amplitudes found above.
2. Static accuracy tests, shown in Table 2. The static errors of the robotic couches generally remained below 0.6 mm. The Perfect Pitch showed the lowest errors in the longitudinal and lateral directions, while the RoboCouch showed the lowest error in the vertical direction.
3. Maximum speed tests, shown in Table 2. The RoboCouch attained the highest speeds in all directions.
4. Maximum acceleration tests, shown in Table 2. The RPSbase attained the highest accelerations in all directions.
5. Time delay tests, shown in Table 2. The Protura showed the smallest time delay of 0.076 s.
6. The last type of tests was the chirp signal test. Figure 3 shows the ratio of the chirp signal amplitudes and the residual motion amplitudes. In the case of the Protura and the Perfect Pitch, the white lines indicate the border above which the maximum speeds and acceleration of the respective couch were exceeded by the chirp signals. The maximum speeds and accelerations of the RoboCouch and the RPSbase were never exceeded for the frequencies and amplitudes investigated. Generally, the residual motion amplitudes were smaller than the chirp signal amplitudes as long as the maximum speeds and accelerations of the couches were respected. However, even under these conditions, the residual motion amplitude increased when the chirp signal frequency increased.

**Table 2:** The requirements and the test results are shown here.

Treatment couch	Motion range (mm)			Static RMS error (mm)			Time delay (s)
	lng	lat	vrt	lng	lat	vrt	
Protura	66	45	44	0.12	0.60	0.56	0.076
Perfect Pitch	66	44	44	0.12	0.12	0.11	0.122
RoboCouch	66	44	44	0.20	0.20	0.04	0.122
RPSbase	66	44	44	0.14	0.19	0.19	0.140
Requirements	60	40	40	0.24	0.24	0.24	—

	Maximum speed (mm/s)			Maximum acceleration (mm/s^2^)		
	lng	lat	vrt	lng	lat	vrt
Protura	20 ± 0	21 ± 0	19 ± 1	49 ± 2	46 ± 2	96 ± 8
Perfect Pitch	81 ± 2	41 ± 0	20 ± 1	96 ± 9	34 ± 5	28 ± 3
RoboCouch	100 ± 0	98 ± 0	100 ± 1	316 ± 3	239 ± 11	231 ± 31
RPSbase	97 ± 1	83 ± 1	79 ± 1	1631 ± 81	2426 ± 83	2261 ± 143
Requirements	60	60	60	80	60	30

Note

The top part shows the results of the motion range tests in the longitudinal (lng), lateral (lat), and vertical (vrt) directions, the static root mean square (RMS) errors for the lng, lat, and vrt directions, as well as the time delays. The bottom part shows the maximum speeds and accelerations in the lng, lat, and vrt directions (mean ± standard deviation).

## 5 DISCUSSION

The application of couch tracking has the potential to reduce the treatment margins, which potentially leads to reduced side effects or an increased disease control of radiation therapy. However, the compensation of tumor motion challenges currently available robotic couches. We have investigated the performance of the Protura, the Perfect Pitch, the RoboCouch, and the RPSbase systems with respect to their motion range, static accuracy, time delay, maximum speeds, maximum accelerations, and tracking performance at sinusoidal motions of various frequencies and amplitudes. Tumor motion traces were characterized by their displacement, frequency, speed, and acceleration. Based on this information, the design for an ideal treatment couch can be derived. The hardware design should be compatible with standard C‐arm gantry systems. The software design should incorporate control algorithms developed specifically for motion compensation.

The traditional use of robotic couches is the correction of the patient setup prior to each treatment. For this use, the main requirement is the static accuracy of the couch, which should be as high as possible. However, due to the limited imaging resolution before treatment, that accuracy may be limited without any negative impact on the correction of the patient setup. The Perfect Pitch, the RoboCouch, and the RPSbase achieve static accuracies of less than 0.24 mm in all three dimensions, while the Protura achieves that accuracy only for the longitudinal direction. Generally, the current robotic couches are designed for this task and fulfill it well.

The new application of robotic couches for couch tracking imposes new requirements, which were derived by analyzing tumor motion data. First, the robotic couches must cover the whole range of tumor displacements. Second, the robotic couches must achieve the same maximum speeds and accelerations as the tumors. As expected, the robotic couches examined all fulfill the first requirement, since for the patient setup, a large motion range is generally beneficial. The RoboCouch and the RPSbase fulfill the speed requirement completely, while the Perfect Pitch does so only for the longitudinal direction and the Protura does not for any direction. Similarly, the acceleration requirement is met by the RoboCouch and the RPSbase, and partly so by the Perfect Pitch, that is, in the longitudinal and vertical direction, and by the Protura in the vertical direction.

The motion range, speed, and acceleration are the necessary requirements for an active motion compensation. However, as Fig. 3 shows, these are not sufficient for a perfect tumor motion compensation. Even if the input motion respects the limits of the motion range, speed, and acceleration, a residual motion is still present. We suspect this nonzero residual motion to result from the control algorithm together with the inertia of the mechanical and drive systems. Any dynamical system with a control algorithm typically lags behind the demanded position, because for the control algorithm to react, a difference has to exist between the actual and the demanded position. The inertia of the mechanical dynamical system here is due to the mass of the robotic couch and the patient. The action of the control algorithm is the force acting on the mechanical system, which results in a lag of the actual position. The control algorithm’s performance is limited additionally by the precision of the sensors and actors in the dynamical system.

The fact that the residual motion of the RoboCouch is small, as evident in Fig. 3, may be due to the system being based on a robot designed for the production industry (e.g., automobiles). Such robots are required to not only be accurate but also to be fast. The small residual motion observed in the RPSbase couch may be explained by its design for the concept of a virtual isocenter.^34^ The mechanical design does not seem to influence the requirements stated above as the RoboCouch and the RPSbase have very different mechanical designs. However, due to its design with robotic arms, the RoboCouch design is less compatible with a linear accelerator in standard C‐arm gantry design than the RPSbase. In Fig. 3, for both the RoboCouch and the RPSbase, the errors appear larger for smaller amplitudes. This may be related to the accuracy of the measurement device, the relative errors of which grow for movement amplitudes, which become small compared to the amplitude of the input motion.

An effective couch‐tracking approach demands very short time lags and delays, which can be decreased in two ways. First, the maximum driving forces are increased while the inertia consisting of robotic couch and patient is decreased. Second, the controller is not only to consider the current demanded position but also its time derivatives speed and acceleration. This approach allows the controller to anticipate future demanded positions. The drawback of increasing the driving forces is the cost, and it could increase bending of the couch as well as introduce issues of mechanical stability. Considering time derivatives as well requires a profound knowledge of the dynamics of the position signal demanded, which to some extent is available for respiratory motion and prostate motion. However, it is likely to be more cost‐effective and would not introduce any mechanical issues. This approach was investigated by combining prediction filters^35^ of respiratory motion and model predictive control methods.^36^

The current robotic couches show that the necessary requirements can be met. The Protura as well as the Perfect Pitch would benefit greatly from faster motors. However, all couches would improve their couch‐tracking performance with adjusted control algorithms even if they meet the necessary requirements already. Such control algorithms should take into account the time derivatives of the tumor motion to reduce the time lag, which is the main cause of residual motion.

The distributions of the tumor motion characteristics showed a substantial asymmetry. The results regarding the speeds and accelerations of the tumor motion are not as reliable as the peak‐to‐peak amplitudes and frequencies because the number of the speed and acceleration data is smaller than the number of amplitude and frequency data. Additionally, the speed and acceleration data were derived by differentiating the position signal by the sampling time, which increases the noise and thus the uncertainty of these signals. As the current results only hold for patient loads of up to 98 kg, further studies could be carried out with patient loads of more than 98 kg.

The control approaches of the robotic couches under consideration here varied widely. However, the impact of the control approach of the robotic couches was negligible when the maximum velocity or the maximum acceleration was tested because in these tests, all robotic couches were set up to move in a minimum time trajectory from one point to another. Also, plausibility checks on the velocity or the acceleration limits could be carried out on the results of the chirp signal tests even though those tests might still be impacted by the software control approach. Nevertheless, the impact would cause results more conservative as well as indicate more potential for performance improvements.

The feasibility of couch tracking was previously examined using a miniaturized model of a couch and a tumor motion simulator.^37^ In Ref. 38, the authors investigated the dynamical parameters such as the speed or the acceleration needed for a robotic couch to successfully compensate tumor motion. However, their results (162 mm/s speed, 887 mm/s^2^ acceleration) were very conservative as our investigation on tumor motion characteristics showed. Various robotic couches were also investigated. The HexaPOD robotic couch was tested with various tumor motion trajectories to be compensated,^9^ or modeled to facilitate the development of the control algorithm for couch tracking.^10^ The ELEKTA robotic couch was tested with various tumor motions.^11^ However, the authors tested only single robotic couches and used a limited number of motion trajectories, whereas in our work, four couches were investigated with a wider range of motion trajectories. More recently, prototypical systems have been developed. A mechanical slider was used to evaluate tracking algorithms for motion compensation.^12^ An industrial robot was combined with a couch plate, and the system’s motion compensation performance was tested.^13^ A robotic couch system was designed explicitly for couch tracking and was tested with various patient‐derived tumor motion trajectories.^14^ The Protura was examined regarding its ability to compensate sinusoidal motion with varying frequencies.^15^ We expanded on those results by using a larger range of amplitudes of the motion trajectories and by including other robotic couches. Tumor motion data were collected in Ref. 39 for the purpose of assessing a motion platform for the quality assurance of motion management techniques. In this paper, the collected data showed smaller values for motion amplitude, velocity, and acceleration than the values found here. The requirements identified in the current study thus comprise the characteristics derived.^39^

The performance results found are limited to the couch itself that is only one part of the couch tracking system. The detection of the tumor position is crucial for an accurate tumor tracking. Furthermore, the motion of the couch can lead to residual motion of the patient’s non‐rigid body.^40^ For a final assessment of the couch tracking approach, the entire system consisting of tumor detection and couch motion has to be tested with patients and their real motion trajectories.

## 6 CONCLUSION

The ideal couch for couch tracking has a motion range of at least 60 mm longitudinally, 40 mm laterally, and 40 mm vertically. The maximum speeds have to be at least 60 mm/s in any direction with the maximum accelerations of 80 mm/s^2^ longitudinally, 60 mm/s^2^ laterally, and 30 mm/s^2^ vertically. These requirements were shown to be fully feasible by the RoboCouch and the RPSbase. However, they can be improved toward the ideal couch with a controller that takes into account not only the current position demanded but also its time derivatives, which can be accomplished using prediction filters. Overall, the static accuracy is a prerequisite for a high dynamic accuracy.

## CONFLICT OF INTEREST

The authors declare that they have no competing interests.
