# Supplementary material for: The ideal couch tracking system—Requirements and evaluation of current systems
Source: J Appl Clin Med Phys. 2019 Sep 19;20(10):152–9. doi: 10.1002/acm2.12731 (PMC6806475; doi:10.1002/acm2.12731)
Supplement: Supplementary file 1 [file ACM2-20-152-s001.pdf]

Supplemental materials for “The ideal couch-tracking system – requirements and evaluation of current systems.”

Authors

Alexander Jöhl[1,2], Stefanie Ehrbar[2,4], Matthias Guckenberger[2,4], Stephan Klöck[2,4], Andreas Mack[5], Mirko Meboldt[1], Melanie Zeilinger[3], Stephanie Tanadini-Lang[2,4], Marianne Schmid Daners[1]

[1] Product Development Group Zurich, Department of Mechanical and Process Engineering, ETH Zurich, Tannenstrasse 3, 8092 Zurich, Switzerland

[2] Department of Radiation Oncology, University Hospital Zurich, Rämistrasse 100, 8091 Zurich, Switzerland

[3] Institute for Dynamic Systems and Control, Department of Mechanical and Process Engineering, ETH Zurich, Sonneggstrasse 3, 8092 Zurich, Switzerland

[4] University of Zurich, Rämistrasse 71, 8006 Zurich, Switzerland

[5] Klinik Hirslanden Zurich, Witellikerstrasse 40, 8032 Zurich, Switzerland

Content

Authors 1
Content 1
Measurement setup of the robotic couches 2
Position measurement device 2

Measurement setup of the robotic couches

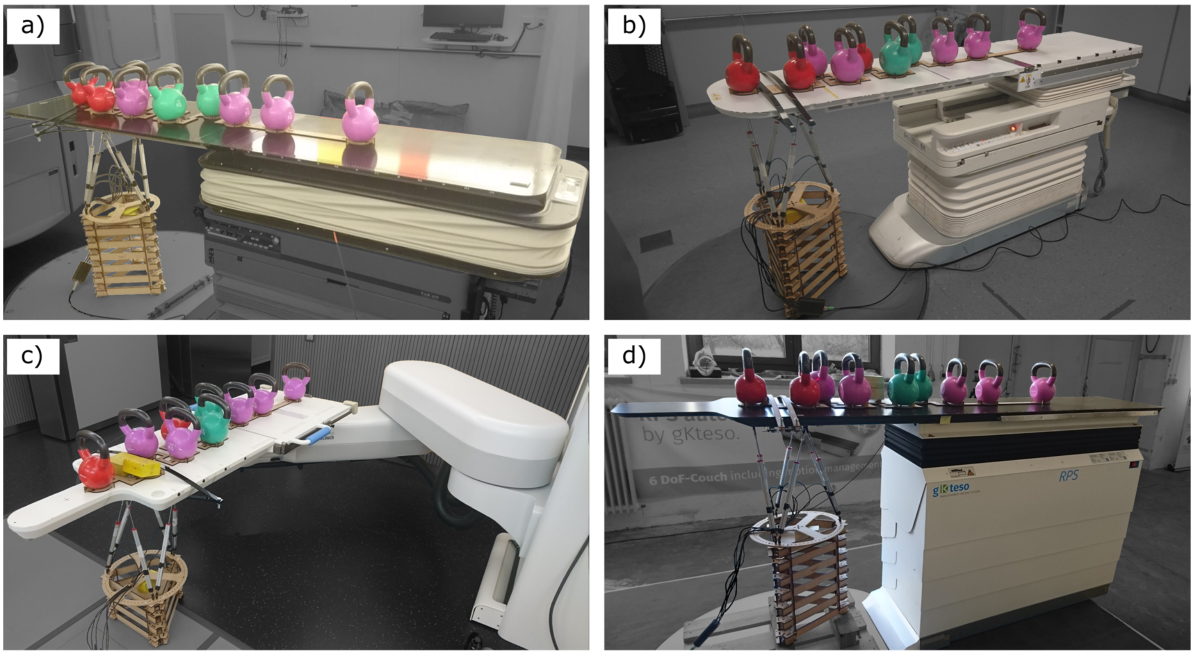

Fig. 1: Setups of the robotic couches for the performance tests, a) shows the Protura (CIVCO Medical Solutions, Kalona, USA), b) Perfect Pitch (Varian Medical Systems, Palo Alto, USA), c) RoboCouch (Accuray Inc., Sunnyvale, USA), and d) RPSbase (gKteso GmbH, Bobingen, Germany). The weights on top imitated a patient with a total weight of 98 kg and were distributed identically for all couches. Red weights were 8 kg, pink weights were 10 kg, and green weights were 12 kg, respectively.

Position measurement device

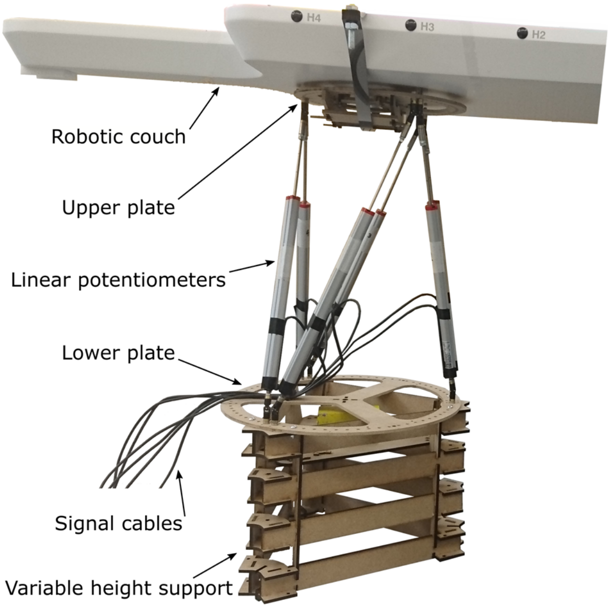

Fig. 2: Measurement device consisted of a lower plate and an upper plate connected by six linear potentiometers in parallel. The upper plate was fixed to the robotic couch. The lower plate was fixed to the support, which was placed on the ground. The height of the support could be varied to accommodate the measurement system for different robotic couches.

The position measurement device consists of six linear potentiometers (Opkon, Istanbul, Turkey), which were arranged in parallel between two plates (Fig. 2). During the performance tests, the measurement device was positioned beneath the couch plate. The lower plate was fixed to the ground, while the upper plate was fixed to the couch. The position and orientation of the upper plate relative to the lower plate correspond directly to the lengths of the potentiometers. Therefore, the signals of the potentiometers could be used to compute the position and orientation of the couch. The analog output signals of the potentiometers were sampled at 500 Hz.

The measurement device was tested with the Hexapod H840.5PD (Physik Instrumente GmbH & Co. KG, Karlsruhe/Palmbach, Germany). The Hexapod moved to points in a three-dimensional grid in a randomized sequence and paused at each point. The differences between the position as given by the H840.5PD and the measured positions as given by the measurement device were considered errors, see Fig. 3. The standard deviation of the translational errors were observed to be 0.12 mm in the longitudinal, 0.13 mm in the lateral, and 0.06 mm in the vertical direction. The rotational errors showed a standard deviation of 0.02° around the longitudinal direction, 0.02° around the lateral direction, and 0.03° around the vertical direction. Fig. 4 shows the errors in the same chronological sequence as the points were reached by the H840.5PD. The figure indicates that no drift or other relevant long-term tendencies were present in the measurement device.

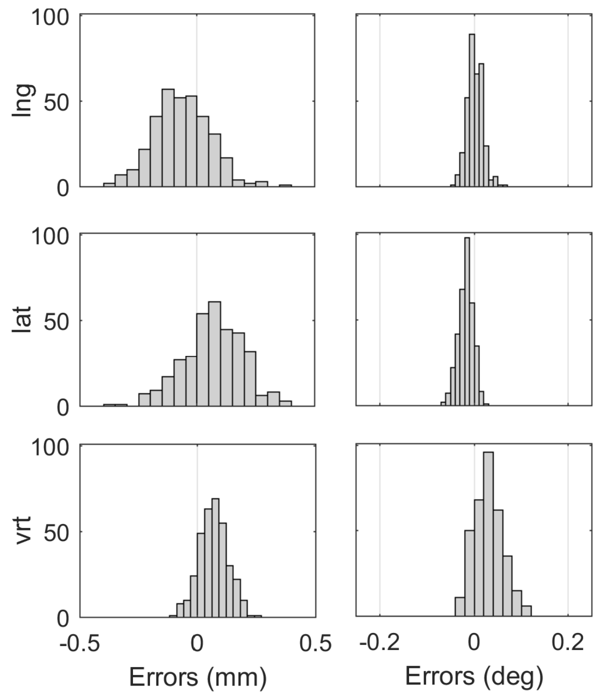

Fig. 3: Histograms of the errors of the position measurement system. The Hexapod H840.5PD (Physik Instrumente GmbH & Co. KG, Karlsruhe/Palmbach, Germany) moved the measurement device into predefined points in a three-dimensional grid and paused at each point. The differences between the actual position as given by the Hexapod and the measured position as given by the measurement device were considered errors. The left panels show the translational errors and the right panels show the rotational errors. The Hexapod only moved translationally.

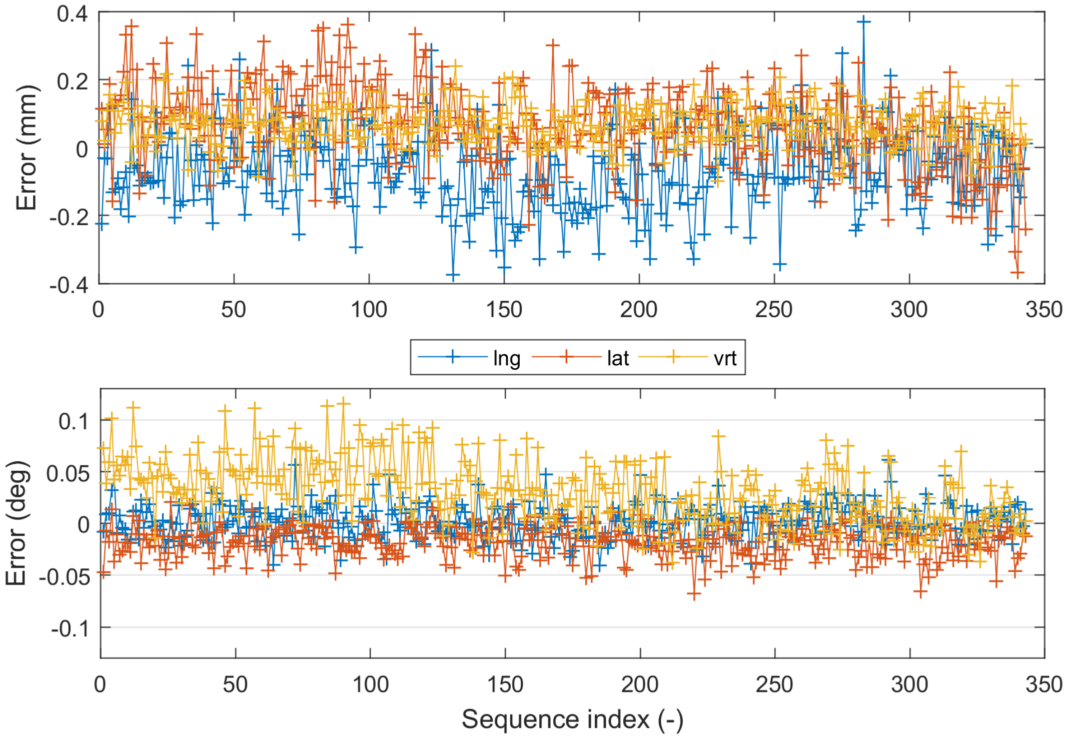

Fig. 4: The errors of the measurement device test using the Hexapod H840.5PD (Physik Instrumente GmbH & Co. KG, Karlsruhe/Palmbach, Germany). The upper panel shows the translational errors, while the lower panel shows the rotational errors. The colors represent the three directions in space, longitudinal (lng), lateral (lat), and vertical (vrt). Each cross represents one point of the three-dimensional grid to which the H840.5PD moved and then paused. The points are shown in the sequence in which they were reached by the H840.5PD.

Maximal time delay required

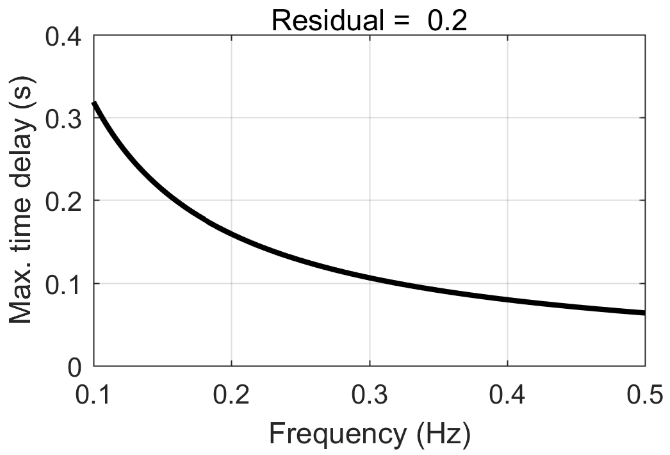

Fig. 5: The black line shows the maximal required time delay for a range of motion frequencies and the given residual motion (normalized by input amplitude). The frequencies varied from 0.1 The required time delay for a given residual motion was determined by modeling the tumor motion as a sinusoidal motion (as in the chirp tests) and the compensation motion as a sinusoidal motion as well but with a phase offset φ.

$$x_{residual}(t) = \sin(2\pi f t) - \sin(2\pi f t - \varphi)$$

The resulting amplitude of the residual motion is $2\sin(\varphi/_2)$. The relationship between the phase offset and the time delay T_{delay} is dependent on the motion frequency $\varphi = T_{delay} 2\pi f$. Therefore, the maximal time delay can be computed by the following formula:

The ideal couch-tracking system

$$T_{delay} = \frac{2}{2\pi f} \arcsin\left(\frac{A_{res,normalized}}{2}\right)$$

In Fig. 5, the results of this formula is shown with $A_{res,normalized} = 0.2$. The maximum time delay varies strongly with the frequency of the tumor motion and decreases when the frequency increases. For a tumor motion frequency of 0.5 Hz a maximum time delay of 64 ms results. However, tumor motion with smaller frequencies can be handled with substantially larger time delay but the same residual motion. Therefore, a time delay of 64 ms is a recommended value, but it is very conservative. For a large number of patients a higher time delay in motion compensation may still result in a beneficial outcome.
